# Renal cell carcinoma with multiple bone metastases effectively treated by a combination of tyrosine kinase inhibitor, robot‐assisted partial nephrectomy, and metastasectomy

**DOI:** 10.1002/ccr3.8482

**Published:** 2024-03-01

**Authors:** Atsuro Sawada, Masashi Takeda, Takayuki Goto, Shusuke Akamatsu, Takashi Kobayashi

**Affiliations:** ^1^ Department of Urology Kyoto University Graduate School of Medicine Kyoto Japan; ^2^ Department of Urology Nagoya University Graduate School of Medicine Nagoya Japan

**Keywords:** metastasectomy, metastatic renal cell carcinoma, multidisciplinary treatment, robot‐assisted partial nephrectomy, tyrosine kinase inhibitor

## Abstract

**Key Clinical Message:**

Maintaining a disease‐free status for a long time in cases of renal cell carcinoma with multiple bone metastases and repeated recurrences is challenging. What matters most in the multidisciplinary approach is the treatment strategy. Although this is a case where multidisciplinary treatment resulted in long‐term CR during the TKI era, the treatment strategy is still relevant now that treatment options have increased.

**Abstract:**

Recent advances in medications, such as immune checkpoint inhibitors (ICIs) and tyrosine kinase inhibitors (TKIs), have improved metastatic renal cell carcinoma (mRCC) outcomes. We report a case of mRCC with bone metastasis that was successfully treated using a multidisciplinary approach. Here, we present a case of a 56‐year‐old man with left renal cancer and large and painful bone metastases at the 11th thoracic vertebrae (Th11). Therefore, a metastasectomy of Th11 was performed. Systemic treatment with TKI, robot‐assisted partial nephrectomy, and metastasectomy were then administered. No recurrence was observed in >2 years. Long‐term disease‐free survival with the TKI‐era multidisciplinary approach in a patient with mRCC remains significant when considering treatment sequences, especially now that drug treatment options—including ICIs—have increased. Treatment strategy and indication and timing of resection of the primary lesion and metastasectomy should be carefully considered in each case.

## Graphical abstract

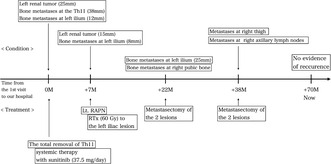

## 1 INTRODUCTION

The availability of new agents, such as tyrosine kinase inhibitors (TKIs) and immune checkpoint inhibitors (ICIs), has greatly improved the treatment outcomes of metastatic renal cell carcinoma (mRCC), allowing us to aim for a disease‐free status through multidisciplinary treatment. However, controversy exists regarding the effectiveness and timing of metastasectomy or nephrectomy for primary lesions during multidisciplinary treatments. Here, we report a case of mRCC with bone metastasis that achieved a disease‐free status through multidisciplinary treatment consisting of systemic therapy with TKI, robot‐assisted partial nephrectomy (RAPN), and metastasectomy.

## 2 CASE PRESENTATION

**FIGURE 1:**
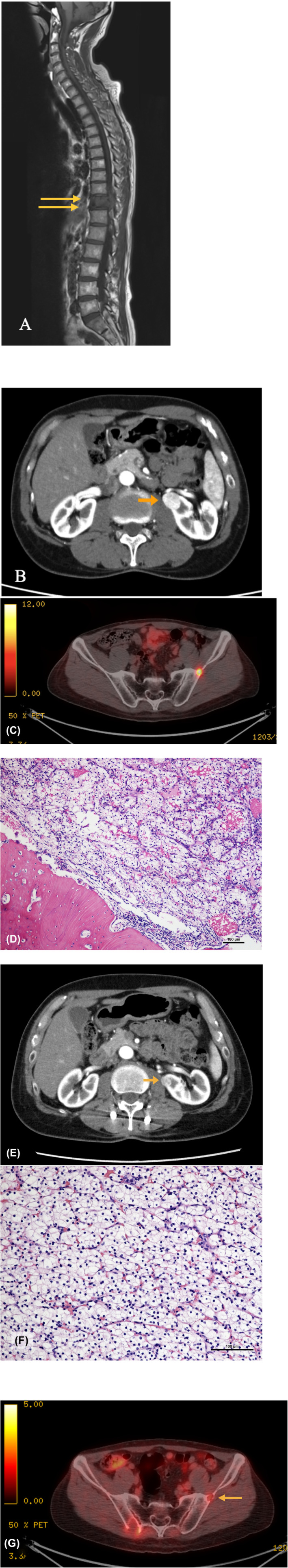
(A) Magnetic resonance imaging at the first visit. Bone metastasis was observed at Th11. (B) Computed tomography at the first visit. A 25‐mm renal tumor suggestive of renal cell carcinoma was found in the proximity of the renal hilum. (C) Positron emission tomography‐computed tomography at the first visit. A 12‐mm bone metastasis was detected in the left ilium. (D) Histopathological images of Th11; hematoxylin and eosin (HE) staining, 100×. The figure shows clear cell carcinoma consistent with renal cancer metastasis. (E) The left renal tumor shrunk after systemic treatment with a tyrosine kinase inhibitor (TKI). (F) Histologic examination of the renal tumor after TKI therapy, HE, 200×. This figure shows clear cell renal cell carcinoma with nests of clear cells surrounded by intricately branching vascular septa. Given that nucleoli are inconspicuous at high magnification, this tumor was diagnosed as Fuhrman grade 2. (G) Bone metastasis in the left ilium after systemic treatment with TKI. The size of the tumor decreased to 8 mm, with very weak accumulation observed on fluorodeoxyglucose‐positron emission tomography.

A 56‐year‐old man visited the orthopedic surgery department of our hospital complaining of low back pain, which occurred mainly during movement and was grade 3 in the classification of low back pain..^1^ At the first evaluation, blood examination revealed no remarkable abnormal findings, such as C‐reactive protein of <0.10 mg/dL, white blood cell count of 3.85 × 10^9^/L, hemoglobin of 14.3 g/dL, lactate dehydrogenase of 147 U/L, albumin of 4.3 g /dL, calcium of 8.8 mg/dL, and estimated glomerular filtration rate (eGFR) of 60.8 mL/min. Though the pain was severe, there was no suspicion of nerve paralysis. His past medical and family history were not noteworthy. Performance status was 0. Computed tomography (CT) and positron emission tomography (PET)‐CT showed a 25‐mm left renal tumor and bone metastases at the 11th thoracic vertebrae (Th11) and left ilium, 38‐ and 12‐mm, respectively (Figure 1A–C).

Given the severity of his back pain and the risk of paralysis, surgical treatment of the thoracic spine was prioritized, which involved the total removal of Th11. Pathological analysis of Th11 revealed clear cell carcinoma (Figure 1D). Thus, a diagnosis of stage cT1aN0M1 left RCC and bone metastasis with International Metastatic RCC Database Consortium (IMDC) intermediate risk was established.

Thereafter, systemic therapy with sunitinib (37.5 mg/day) was started. Because the ICI combination therapy was not yet approved at that time, sunitinib, which was recommended as the first‐line treatment for intermediate‐risk cases, was used. Despite grade 2 hand–foot syndrome and general fatigue, nine courses for 6 months were completed, which decreased the size of the tumors in the left kidney and left ilium to 15 and 8 mm, respectively, with no other obvious metastatic lesions (Figure 1E).

We performed RAPN for the primary lesion with a margin of at least 5 mm. Pathological findings showed residual 15 mm × 12‐mm viable cancer cells of Fuhrman grade 2, with marked inflammatory cell infiltration in the surrounding renal parenchyma (Figure 1F). Pre‐ and postoperative renal function showed only slight change with an eGFR of 66 and 59 mL/min/1.73 m^2^, respectively. Although the left iliac lesion had shrunk to 8 mm and the fluorodeoxyglucose (FDG) uptake on PET‐CT had also decreased, radiotherapy (60 Gy) to the left iliac lesion was selected as an additional treatment (Figure 1G). After these treatments, the patient was surgically in a state of complete response (CR) with no evaluable lesions. The use of bone‐targeted agents was considered as well. However, the size of the bone lesions was so small and asymptomatic that these agents were not used and the patient was followed up without additional systemic treatment.

**FIGURE 2:**
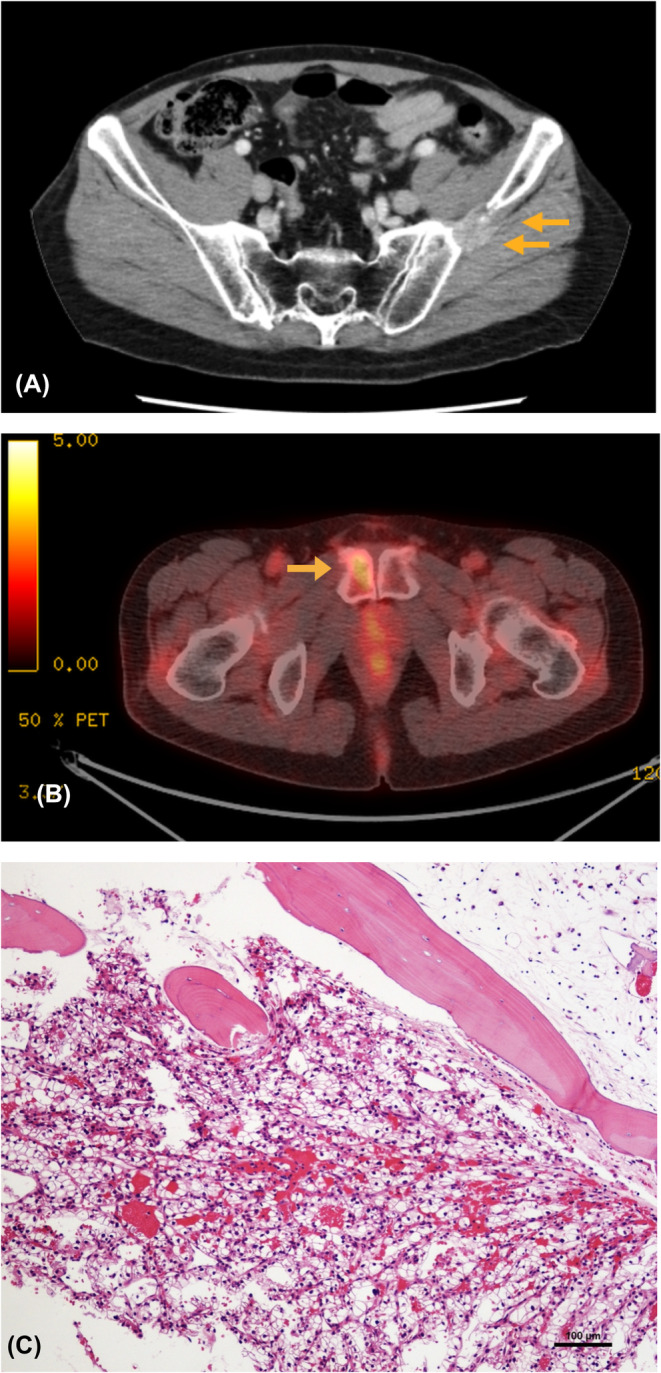
(A) Bone metastasis of the left ilium upon re‐enlargement. (B) Fluorodeoxyglucose accumulation in the right pubic bone on positron emission tomography‐computed tomography. (C) Pathology of the left iliac lesion; hematoxylin and eosin staining, 100×. The figure shows metastases of renal carcinoma, which invaded while destroying the trabeculae of the ilium.

Although the left iliac lesion remained small for 1 year and 3 months, it grew and enlarged to 25 mm (Figure 2A). FDG‐PET also showed slight uptake in the right pubic bone, indicative of bone metastasis, with no other metastatic lesions (Figure 2B). Since no bone metastases caused bone destruction or pain, and other lesions were observed, a bone‐modifying agent was not administered. Instead, both lesions were removed through metastasectomy. Pathological findings were consistent with metastasis of RCC (Figure 2C).

**FIGURE 3:**
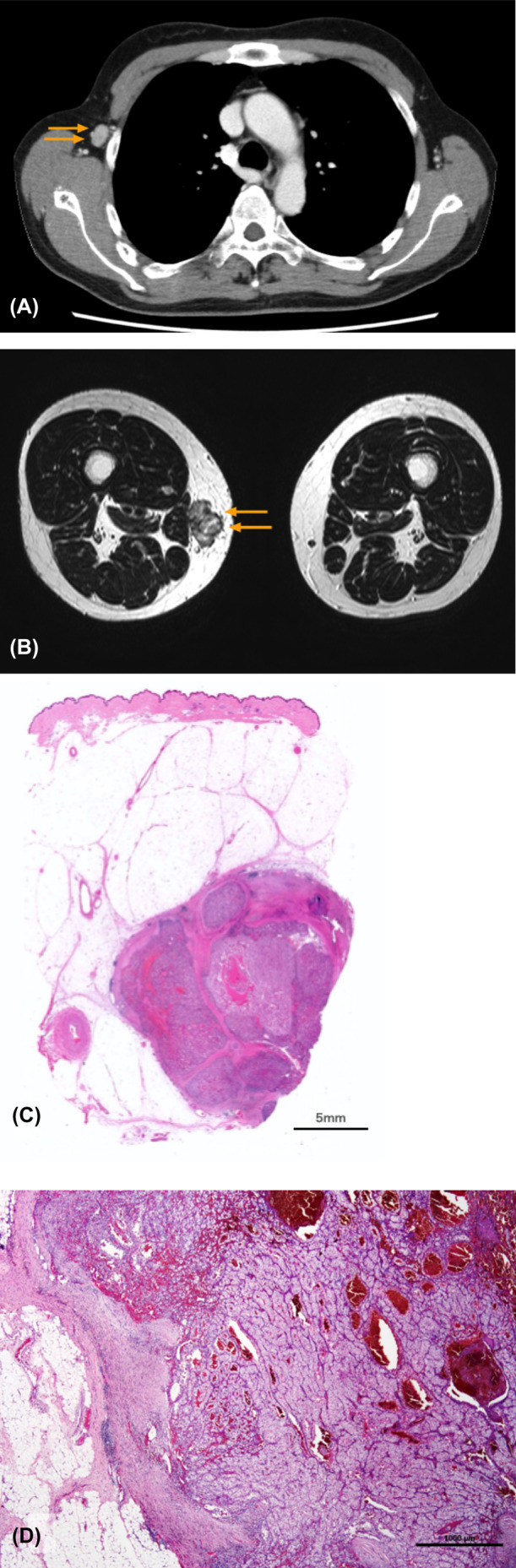
(A) Computed tomography showing enlarged lymph nodes in the right axilla. (B) Magnetic resonance imaging showing a subcutaneous tumor in the right thigh. (C) Right axillary lymph node; hematoxylin and eosin staining, 20×. Lymph nodes were destroyed by the metastasis of the renal tumor. (D) Subcutaneous tumor of the right thigh; hematoxylin and eosin staining, loupe. Spherical metastases are seen within the subcutaneous adipose tissue.

**FIGURE 4:**
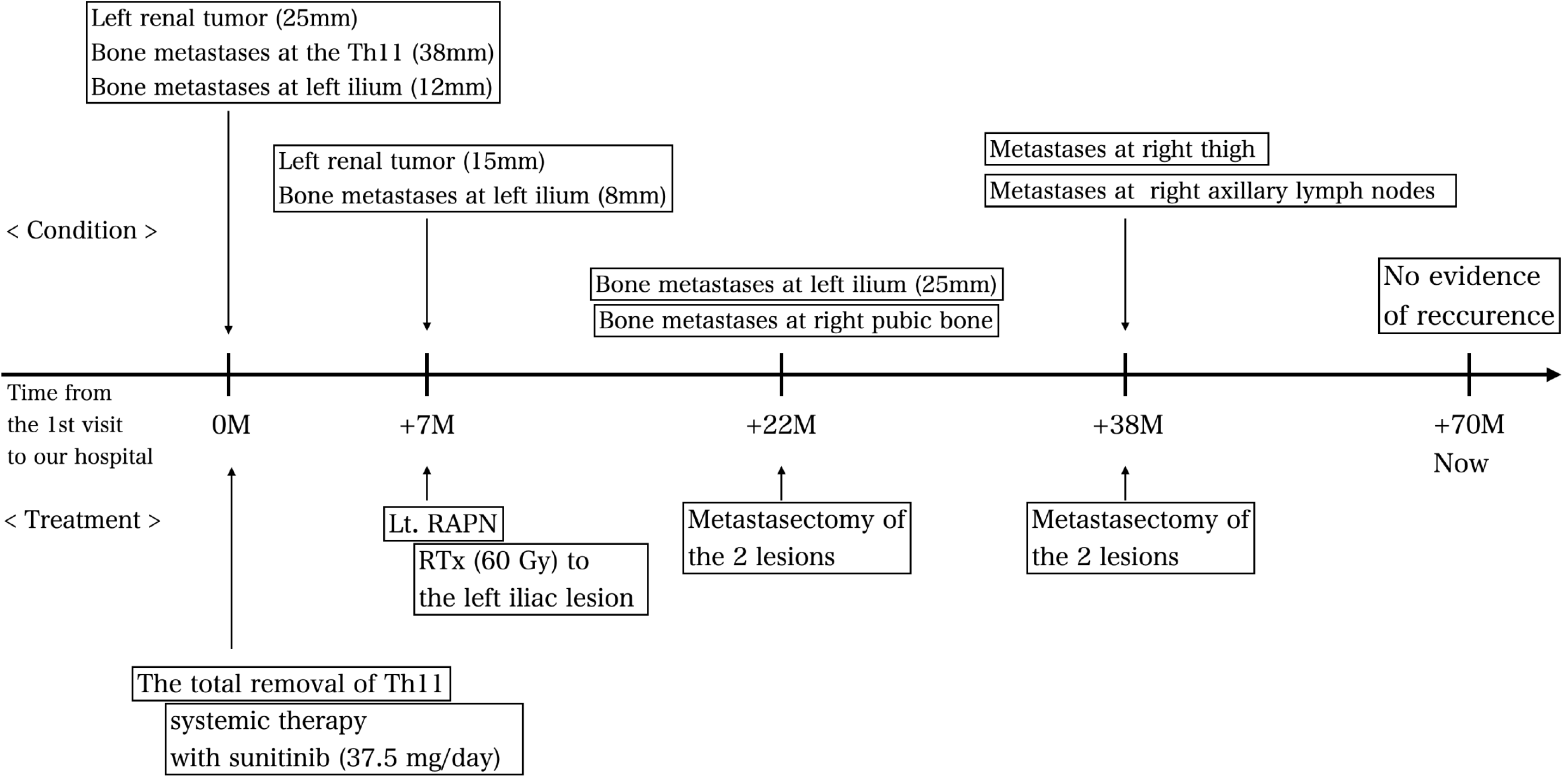
Timeline that shows the relationship between the clinical condition and the course of treatment from the first visit to today.

After 1 year and 4 months, two new metastatic lesions, each 15 mm in size, were found in the right medial thigh and right axillary lymph nodes, with no other metastatic lesion recognized (Figure 3A, B). They were resected via orthopedic and breast surgery, and both pathological diagnoses were consistent with metastasis of RCC (Figure 3C, D). The patient was followed up without additional treatment. No apparent metastasis or recurrence had been observed for over 2 years since the last metastasectomy. Figure 4 shows the timeline to date.

## 3 DISCUSSION

The bone is the second most common site for RCC metastasizing. A study involving 2027 patients with mRCC treated with TKI showed that patients with and without bone metastases had a median overall survival (OS) of 14.9 and 25.1 months and median time‐to‐treatment failure (TTF) of 5.7 and 7.6 months, respectively. In cases with bone‐only metastasis, the TTF was comparable to that without bone metastasis (8.1 months); however, OS was still poor (16.8 months).^2^ Therefore, TKI monotherapy is not sufficient for the treatment of mRCC with bone metastasis. Currently, ICI + ICI or TKI + ICI is recommended as the first‐line treatment of mRCC. Compared with sunitinib alone, these treatments have been found to be more effective, with better benefits in progression‐free survival (PFS), OS, and objective response rate. With these treatments, CR can be expected even with medication alone; however, even if partial response and stable disease remain, surgical CR can be achieved by multidisciplinary treatment using surgical resection and radiotherapy for residual tumors, as in this case.

The role of local treatment of renal tumors during multimodal therapy remains controversial. Several negative opinions have been raised regarding cytoreductive nephrectomy (CN) in the TKI era, given that sunitinib alone was not inferior to sunitinib + CN in the CARMENA study. A study on 46 patients treated with TKI after CN by Barbastefano showed that the fractional percentage of the tumor volume was an important factor in prolonging the PFS.^3^ Another retrospective study comparing radical nephrectomy and PN after systemic therapy showed that PN prolonged OS when the tumor was ≤4 cm.^4^ Therefore, in the current case, performing RAPN after TKI was preferred over CN. High‐level evidence regarding CN is not available for ICI combinations. In accordance with the CARMENA and SURTIME data, patients with IMDC intermediate‐ and poor‐risk mRCC with their primary tumor in place should be treated with upfront ICI combinations. In patients with a clinical response to ICI combinations, a subsequent CN may be considered.

Whether metastasectomy should be performed for bone metastases is also controversial. In a study on 114 patients with mRCC and bone metastasis, Du reported that TKI + metastasectomy and TKI alone resulted in a median OS of 31.8 and 9.9 months, respectively.^5^ According to the EAU guideline, retrospective comparative studies consistently pointed toward the benefit of complete metastasectomy in patients with mRCC in terms of OS, cancer‐specific survival, and delay of systemic therapy. These findings suggest that metastasectomy may have promoted clinical outcomes, including survival in the current case. Stereotactic radiotherapy is also an option to consider for bone lesions; however, in this case, the iliac lesion was re‐progressed despite 60 Gy radiotherapy, and this renal cancer might be less sensitive to radiation therapy. Therefore, we prioritized metastasectomy for subsequent bone recurrence.

The “vicious cycle” hypothesis has explained how renal cancer cells interact with the bone microenvironment to cause bone destruction and tumor growth. However, evidence has suggested that cancer cells in the bone marrow initially enter a dormancy state, followed by the start of a vicious cycle and cell proliferation after a considerable period.^6^ Thus, given that clinically identifying microbone metastasis is time‐consuming, the resection of all visible bone metastases at some point could have contributed to the prognosis of the current case.

As a limitation, it should be added that the course of this case is not necessarily universal because of the wide range of characteristics of renal cancer.

## 4 CONCLUSION

We herein report a case of mRCC with bone metastasis that was successfully treated with a multidisciplinary approach combining TKI, metastasectomy, and RAPN. Treatment strategy, including the indication and timing of resection of the primary lesion and metastasectomy, should be carefully considered in each case.

## AUTHOR CONTRIBUTIONS

**Atsuro Sawada:** Writing – original draft; writing – review and editing. **Masashi Takeda:** Data curation. **Takayuki Goto:** Supervision. **Shusuke Akamatsu:** Supervision. **Takashi Kobayashi:** Supervision.

## FUNDING INFORMATION

This research did not receive any specific grant from funding agencies in the public, commercial, or not‐for‐profit sectors.

## CONSENT

Written informed consent was obtained from the patient to publish this report in accordance with the journal's patient consent policy.

## Supporting information


Data S1.

## Data Availability

Data available on request from the authors.
