# Supplementary material for: Renal cell carcinoma with multiple bone metastases effectively treated by a combination of tyrosine kinase inhibitor, robot‐assisted partial nephrectomy, and metastasectomy
Source: Clin Case Rep. 2024 Mar 1;12(3):e8482. doi: 10.1002/ccr3.8482 (PMC10907337; doi:10.1002/ccr3.8482)
Supplement: Supplementary file 1 — Data S1. [file CCR3-12-e8482-s001.pdf]

Author First, Quality First
www.enago.jp

localize to globalize
www.ulatus.jp

we are all ears
www.voxtab.jp

CERTIFICATE OF EDITING

This is to certify that the paper titled Renal cell carcinoma with multiple bone metastases effectively treated by a combination of TKI, RAPN, and metastasectomycommissioned to us by Atsuro Sawada has been edited for English language, grammar, punctuation, and spelling by Enago, the editing brand of Crimson Interactive Pvt. Ltd under Advance Editing B2C.

✓ ISO 17100:2015
Translation Service Providers

✓ ISO 27001:2013
Information Security Management System

✓ ISO 9001:2015
Quality Management System

Issued by:

Enago, Crimson Interactive Pvt. Ltd.
1001, Techniplex - II, S. V. Road,
Goregaon (W), Mumbai 400062, India.
Phone: 03-5050-5374
Fax: 03-4496-4934

Disclaimer : The intent of the author's message has been preserved during the editing process. The author is free to accept or reject our changes in the document after reviewing our edits. This certificate has been awarded at the time of sharing the final edited version (full file or sections of the file) with the author. Enago does not bear any responsibility for any alterations done by the author to the edited document post 1 9月 2022.

**Japan**	www.enago.jp, www.ulatus.jp, www.voxtab.jp	**Russia**	www.enago.ru
**Taiwan**	www.enago.tw, www.ulatus.tw	**Arabic**	www.enago.ae
**China**	www.enago.cn, www.ulatus.cn	**Turkey**	www.enago.com.tr
**Brazil**	www.enago.com.br, www.ulatus.com.br	**S. Korea**	www.enago.co.kr
**Germany**	www.enago.de	**Global**	www.enago.com, www.ulatus.com, www.voxtab.com

About Crimson:
Crimson Interactive pvt ltd is one of the world's leading academic research support services. Since 2005, we've supported over 2 million researchers in 125 countries with their publication goals.

JAT
American Translators Association
Society for Scholarly Publishing
American Medical Writers Association
European Association of Science Editors EASE
